# Supplementary material for: A unified analytic framework for prioritization of non-coding variants of uncertain significance in heritable breast and ovarian cancer
Source: BMC Med Genomics. 2016 Apr 11;9:19. doi: 10.1186/s12920-016-0178-5 (PMC4828881; doi:10.1186/s12920-016-0178-5)
Supplement: Additional file 1: — Supplementary Methods. (DOCX 243 kb) [file 12920_2016_178_MOESM1_ESM.docx]

**SUPPLEMENTARY METHODS**

**Design of Tiled Hybridization Capture Reagent for BRCA Gene Panel**

Probe sequences within single copy intervals [1] in *ATM, BRCA1, BRCA2, CDH1, CHEK2, PALB2,* and *TP53* were selected using PICKY 2.2 software [2]; settings set to 65ºC T_m_, 30-70% GC content, 5 probes per sequence, 20 nt maximum overlap, all other settings default. PICKY will only report a maximum of 5 oligos per sequence analyzed. Therefore, gene sequences were split into 100 nt segments, overlapping by 50 nt. Probes were designed for both the forward and reverse strand of each gene. These overlapping and opposite-stranded sequences were run through PICKY separately; as the program would remove identical probes selected from two sequence segments (including probes which are reverse compliments of each other). This method helps result in significant probe overlap, which leads to a more efficient capture. If regions were lacking probes due to high/low %GC, the process was repeated with expanded GC settings (20% minimum or 80% maximum) and probes over those regions were added to the initial probe file. A Perl script entitled “Amalgamated-Post-Picky-Program” was written to perform 4 tasks: 1) MPI-BLAT (through Shared Hierarchical Academic Research Computing Network or SHARCNET [3]) each PICKY-selected oligo to the transcriptome and eliminate any which match elsewhere (< 1 hour on 16 nodes), 2) Eliminate redundant probes (i.e. removal of a smaller probe overlapped by a larger probe on the same strand), 3) Reduce highly overlapped regions by eliminating one of two near identical probes that differ by a 1 nt shift, and 4) Generate a BED genome browser track of the accepted oligos for visual evaluation of the coverage of the generated tiling array oligos.

**Generating, Cleaving, and Purifying Tiled BRCA Microarray Oligos**

Primer binding sites were added to each end of the designed capture oligos (5’ ATCGCACCAGCGTGTN_36-70_CACTGCGGCTCCTCA). The selected sequences were then synthesized onto two cleavable 12K microarray chips using a Combimatrix B3 CustomArray Synthesizer (CustomArray, Inc., Bothell, WA) in our laboratory, requiring approximately 48 hours. Forward and reverse strand oligos were placed on separate chips to avoid cross-hybridization between complementary strands, which could reduce capture efficiency.

The cleavable microarrays were treated with concentrated (14.5N) ammonium hydroxide at 65ºC for 4 hours. This served to break the sulfonyl-amidite bond linking the oligonucleotide to the microarray. The base was cooled, transferred into a microcentrifuge tube, and placed into a speed-vac for 1 hour at 65ºC. The resulting pellet was resuspended into 100uL of 1x TE buffer, and was then purified using a MicroSPIN DNA column (#11814419001, Roche, Indianapolis, IN). Purified oligos were then amplified by conventional PCR (25 cycles) using Kapa HiFi DNA Polymerase (#KK2602, KapaBiosystems, Wilmington, MA) (forward 5-CTGGGAATCGCACCAGCGTGT-3; reverse 5-CGTGGATGAGGAGCCGCAGTG-3).

The PCR product was purified using a Qiagen MinElute PCR Purification Kit (#28006, Qiagen, Valencia, CA) and then amplified again (25 additional cycles) using a forward primer with an SP6 promoter-binding site (5-CATACGATTTAGGTGACACTATAGAAATCGCACCAGCGTGT-3). Biotin-labelled RNA bait was generated from this product using a MAXIscript SP6 *in vitro* transcription kit (#AM1310, Ambion, Carlsbad, CA) with a UTP to biotin-16-UTP (#11388908910, Roche) ratio of 4 to 1.

**Sample Preparation, Library Preparation, and Oligo Capture for Sequencing**

Genomic DNA (gDNA) was previously extracted from whole blood using the MagNA Pure Compact Nucleic Acid Isolation kit I (#03730964001, Roche) and stored in 1x low TE buffer. Samples with inadequate available gDNA (< 3 μg) were whole genome amplified using the Illustra GenomiPhi V2 DNA Amplification Kit (#25-6600-30, GE Healthcare, Little Chalfont, UK). The gDNA was diluted to 100 ng/μL in a volume of 51 μL for S220 Focused-ultrasonicator (Covaris, Woburn, USA) shearing (150-300 nt fragments generated with the following settings: Time 120 sec, Duty cycle 10%, Intensity 5, and Cycles per burst 200).

The sheared samples were prepared using KAPA Biosystems Standard (KK8200, Kapa Biosystems) and High Throughput (KK8234, Kapa Biosystems) Library Preparation kits, following the manufacturer’s protocol. Standard Illumina paired-end and multiplex adapter oligos and primers (sequences provided by Illumina) were purchased from IDT (Coralville, IA). Adapters were hybridized together by mixing two oligos to a final concentration of 100 μM each, heating to 95ºC for 5 minutes on a thermocycler (Mastercycler pro, Eppendorf) and gradually cooling 0.1ºC/sec to 4ºC. Samples being treated were initially purified using Qiagen MinElute PCR purification kits and Sigma GenElute gel purification kits (#NA1111, Sigma, St. Louis, MO). Sample loss was greatly decreased by switching to DNA-binding Agencourt Ampure XP beads (#A63880, Beckman Coulter, Brea, CA), using the protocol described in Fisher et al. (2011), which allows the re-use of beads by the rebinding of DNA using a 20% polyethylene glycol (PEG) in a 2.5M NaCl solution and avoids gel extraction and column purification steps [4]. The switch to Ampure beads allowed for the automation of sample preparation using a Beckman Coulter BioMek FX workstation, increasing sample throughput to 32 samples processed simultaneously (end-repair, A-tailing, and adaptor ligation steps were performed on the BioMek FX along with all wash steps; PCR steps were performed separately in a thermocycler). The amplified library samples were then reduced to a volume of 6.8 μL using a SpeedVac concentrator prior to genomic capture.

Genes of interest were enriched with Tiled *BRCA* RNA bait, following a modified version of the hybridization selection protocol from Gnirke et al. (2009) [5]. Modifications which increased coverage include: a) an increase in sample quantity from 0.5 μg to half of the library prepared per capture (1 to 2 μg depending on sample prep yield), b) an RNA bait increase from 0.5 to 1.5 μg and, c) an increase in the quantity of M-280 streptavidin Dynabeads (#1205D, Invitrogen, Carlsbad, CA) from 50 μL to 75 μL to account for this increase in RNA bait concentration. Genomic sequences in each library sample were captured in two separate solutions, one for each strand of RNA bait (which were pooled at the end of the procedure). The capture was then incubated at 65ºC for 66-72 hours, in a thermocycler with the heated lid set to 80°C (to help reduce volume loss due to evaporation). Forward and reverse strand capture reactions for the same sample were then purified with streptavidin beads and mixed on a nutator (Adams Nutator, #1105, Clay Adams, Franklin Lakes, NJ) for 30 minutes at room temperature. Use of freshly transcribed RNA bait also greatly improved coverage values. These improvements enabled multiplexed sequencing (4 samples per lane), where the index was added during the post-hybridization amplification using standard Illumina Multiplex PCR Primers (#2, 4, 6, 7; chosen for index dissimilarity). After post-hybridization amplification, remaining primers were removed by Ampure bead purification. DNA samples were then quantified using qPCR following the protocol outlined by KAPA Library Quantification Kit for Illumina Platform (#KK4824, KAPA Biosystems). Samples were then pooled (if multiplexing), and treated to standard Illumina paired-end sequencing on a Genome Analyzer IIx. Read length was increased from 36x36 (sequencing batch 1 and 2) to 50x50 (batch 3) and finally to 70x7x70 (batch 4, 5 and 6) to maximize overall coverage.

**Position Weight Matrix Generator (PoWeMaGen)**

We have developed an information weight matrix generator designed to automatically create position weight matrices (PWMs). These PWMs are used to accurately predict and localize transcription factor binding sites (TFBSs) from genome-scale epigenetic data (i.e. ChIP-seq). The PoWeMaGen software engine, in its current implementation, is set to run exclusively on SHARCNET. The script, however, can easily be converted to run on different Linux-based platforms. The engine, outlined in **Figure SM1** below, has three primary sections: Preliminary file processing, ChIP-seq data filtering, and the execution of the Bipad.

The PoMaWeGen model analyzer program acts as an intermediary in the model generation pipeline, beginning by processing of input, output, and run-time-dependent files. This input includes the epigenetic data, and if selected, the DNAse hypersensitivity data set (track: EncodeRegDnaseClusteredV3). The epigenetic data was filtered by intersection using intersectBed, from the BEDtools package [6]. In this study, available ChIP-seq and CLIP-seq (Cross-Linking ImmunoPrecipitation) data was intersected with DNAse I hypersensitivity tracks for only TFs.

Model building is based on Bipad [7], an algorithm we previously published to minimize entropy across a set of unaligned sites. Bipad is run with biologically-inspired parameters, i.e. whether the site is known to be homogenous or bipartite, if bipartite, allowing for defined range of gap lengths separating half sites. Each sequence may, but is not required, to contain one binding site. This program employs the sq framework to run jobs in parallel. The parts of this program that run in parallel perform the vast majority of computation, and are perfectly parallel. This program spawns a job for each model that it generates. Only one job is spawned if UII is not employed (i.e. if confined to a specific motif length). Each spawned job runs a single instance of Bipad, which comprises the vast majority of the computational load for this step.

Epigenetic input is commonly provided as interval data, but Bipad requires input as sequence. PoWeMaGen converts the interval data to sequence using the reference genome (Encyclopedia of DNA Elements [ENCODE] [8]) and the BEDtools program fastaFromBed. Then a custom java program called Fasta2Bipad.java converts this fasta file to the format required by Bipad. Finally, the engine executes Bipad, with user-selected arguments which specify, among other things, whether the model is homogenous or bipartite, use ZOOPS (Zero or One Occurrence Per Sequence) or OOPS (One Occurrence Per Sequence), whether to consider one or both reading strands, estimated motif length, and the number of Monte Carlo cycles required.

In instances where the length of the site has not been well defined in published data, the Unit Information Increment Index heuristic, which maximizes the information density across the binding site as a function of binding site length [9] was be used to compute the length. This algorithm chooses a range of motif lengths, considers DNA helical periodicity of 10.6 base pairs, and varies these lengths based upon the result of UII computations.

**Selecting TFs for Model Building**

TF ChIP-seq data (track: wgEncodeRegTfbsClusteredV2) and DNaseI Hypersensitivity Clusters in 125 cell types from ENCODE were downloaded and intersected using the Galaxy Browser [10–12], to select for DNA regions accessible to TFs for binding. We then extracted genomic intervals from 10 kb upstream of the transcriptional start site, up to the end of intron 1, for each of the 7 genes. We identified 146 TFs with evidence for binding to the promoters of the genes in our panel.

To assess the expression of these TFs in breast and/or ovarian tissues, expression levels were determined using the Illumina BodyMap through Ensembl (https://www.ebi.ac.uk/gxa/experiments/E-MTAB-513; expression values are available in **Supplemental Table S1**). All TFs (of which IT models were built) were found to be expressed either in normal breast or in ovarian tissues (where FPKM > 0.1). However, certain TFs were expressed at low levels in GATA-1, HNF-3β, HNF-4α (FPKM < 0.1 in breast tissues) and PAX-5 (FPKM < 0.1 in ovarian tissues).

As the Illumina BodyMap expression values were for normal tissues, the expressions of these TFs in context (breast and ovarian cancer cells) were obtained through the cBioPortal (http://www.cbioportal.org/). In these data sets, we find the expression of HNF-3β and PAX-5 have strong variability between breast cancer tissue samples (23.6 ± 60.5 and 2.1 ± 82.4, respectively), while GATA-1 and HNF-4α is less variable, although some cancers were detected to have significant expression (maximum expression values of 32.9 and 163.7, respectively).

**Generating SNPfold Input**

A script was written to retrieve 100 nt of mRNA sequence up- and downstream of the variant of interest while altering the sequence based on any other variants found in the region during sequencing (phase was ignored). This was used as the input data for SNPfold. Only transcribed sequence was included (if variant was < 100 nt from start/end of transcription). For long-range effects, the variants were evaluated against the entire UTR they resided in (using the reference sequence; patient-specific variants were not included).

To determine the background rate of prioritizing variants flagged by this method, we used SNPfold v1.0 (running over RNAfold v1.8.5) to get predictions of structure change for dbSNP 144 SNVs in 5' and 3'UTRs of the 7 genes that were focused on in this study (excluding those already flagged in **Table 3**). We used the 'refgene' table in hg19 coordinates (obtained 12/15/2015) to pull down UTR sequences as defined by refseq annotation, and for each gene we used the UTRs that correspond to the canonical transcript as defined by the 'knownCanonical' table in UCSC genome (obtained 1/28/2016). Additionally, we note that all variants we analyzed do not fall in the coding region of any refseq transcript, and that any variants that did were excluded from SNPfold analysis. For all sequences less than 2000 nt in length we obtained accurate p-values for each qualifying dbSNP 144 SNV, as described in Halvorsen et al. 2010. Following the standards originally described in running of the SNPfold algorithm, for sequences that are longer 2000 nt, we use estimated p-values in SNPfold. This resulted in us being able to obtain accurate p-values for variants in all 5'UTR sequences, and 5 out of 7 3'UTR sequences. For the other 2 3'UTR sequences (*ATM* and *CDH1*) we estimated the p-value via methods previously described.

**Generation of RNA Binding Protein Models from RBPDB and CISBP-RNA**

PWMs for 156 RBPs were downloaded from the RBPDB and CISBP-RNA [13–15]. PWMs containing frequencies were converted into information weight matrices (N = 147). Binding sites for factors with low expression in normal breast tissues (N= 59) based on the GTEx database [16] RPKM < 10 were removed. We also eliminated binding sites for factors with highly variable expression among the 57 available breast tissue samples (where median RPKM < 3 standard deviations; N=11). All UTR variants were then analyzed with models for the remaining RBPs (N=76).

Models for the following factors were used to determine variant effect on RBP binding sites: A2BP1, ANKHD1, ANKRD17, BRUNOL4, BRUNOL5, BRUNOL6, BX511012.1, CIRBP, CSDA, DAZAP1, EIF4B, ELAVL1, FMR1, FUS, FXR1, FXR2, G3BP2, HNRNPA1, HNRNPA2B1, HNRNPA3, HNRNPC, HNRNPF, HNRNPH1, hnRNPK, HNRNPL, hnRNPLL, HNRNPR, HNRPDL, KHDRBS1, KHSRP, MATR3, MBNL1, NCL, NONO, NOVA2, PABPC1, PABPC4, PABPN1, PCBP1, PCBP2, PCBP4, PSPC1, PTBP1, PTBP2, PUM2, RALY, RBFOX2, RBM28, RBM42, RBM5, RBM6, RBM8A, RBMS1, RBMX, ROD1, SAMD4B, SART3, SF3B4, SFPQ, SNRNP70, SNRPA, SNRPB2, SRSF1, SRSF10, SRSF2, SRSF4, SRSF6, SRSF7, SRSF9, SYNCRIP, TAF15, TARDBP, TIA1, U2AF1, U2AF2, ZCRB1, ZNF638.

Models were not built for the following models because only blank files were provided by CISBP-RNA (N=9): ELAVL4, FUBP1, G3BP1, HNRNPAB, KHSRP, NOVA1, PUM1, PUM2, SRSF5, TRA2B, ZRANB2 (KHSRP and PUM2 models present in RBPDB, therefore not included in count).

Models were not built for the following factors due to variable expression between the 57 available breast tissue samples (where median RPKM < 3 standard deviations) (N=11): ACO1, MBNL2, PABPC1L, RBM3, RBM38, SRSF3, YBX1, YTHDC1, ZFP36, ZFP36L1, ZFP36L2.

Models were not built for the following factors due to low or extremely variable expression in normal breast tissue (N=59): A1CF, CELF3, CNOT4, CPEB2, CPEB3, CPEB4, EIF2S1, ELAVL2, ELAVL3, ENOX1, ENOX2, ESRP1, ESRP2, HNRNPA1L2, HNRNPCL1, HNRNPH2, IGF2BP1, IGF2BP2, IGF2BP3, KHDRBS2, KHDRBS3, LIN28A, LIN28B, MBNL3, MEX3B, MEX3C, MEX3D, MSI1, MSI2, PABPC3, PABPC5, PABPN1L, PCBP3, PPRC1, QKI, RBFOX3, RBM24, RBM4, RBM41, RBM45, RBM46, RBM47, RBM4B, RBMS2, RBMS3, RBMXL1, RBMXL2, RBMXL3, RBMY1A1, RBMY1B, RBMY1D, RBMY1E, RBMY1F, RBMY1J, SAMD4A, SRSF12, STAR, YBX2, ZC3H10, ZFP36.

**SUPPLEMENTARY METHODS REFERENCES**

1. Dorman SN, Shirley BC, Knoll JHM, Rogan PK: **Expanding probe repertoire and improving reproducibility in human genomic hybridization**. *Nucleic Acids Res* 2013, **41**:e81.

2. Chou H-H, Hsia A-P, Mooney DL, Schnable PS: **Picky: oligo microarray design for large genomes**. *Bioinforma Oxf Engl* 2004, **20**:2893–2902.

3. **Shared Hierarchical Academic Research Computing Network (SHARCNET)** [https://www.sharcnet.ca/my/front/]

4. Fisher S, Barry A, Abreu J, Minie B, Nolan J, Delorey TM, Young G, Fennell TJ, Allen A, Ambrogio L, Berlin AM, Blumenstiel B, Cibulskis K, Friedrich D, Johnson R, Juhn F, Reilly B, Shammas R, Stalker J, Sykes SM, Thompson J, Walsh J, Zimmer A, Zwirko Z, Gabriel S, Nicol R, Nusbaum C: **A scalable, fully automated process for construction of sequence-ready human exome targeted capture libraries**. *Genome Biol* 2011, **12**:R1–2011–12–1–r1. Epub 2011 Jan 4.

5. Gnirke A, Melnikov A, Maguire J, Rogov P, LeProust EM, Brockman W, Fennell T, Giannoukos G, Fisher S, Russ C, Gabriel S, Jaffe DB, Lander ES, Nusbaum C: **Solution Hybrid Selection with Ultra-long Oligonucleotides for Massively Parallel Targeted Sequencing**. *Nat Biotechnol* 2009, **27**:182–189.

6. Quinlan AR, Clark RA, Sokolova S, Leibowitz ML, Zhang Y, Hurles ME, Mell JC, Hall IM: **Genome-wide mapping and assembly of structural variant breakpoints in the mouse genome**. *Genome Res* 2010, **20**:623–635.

7. Bi C, Rogan PK: **Bipartite pattern discovery by entropy minimization-based multiple local alignment**. *Nucleic Acids Res* 2004, **32**:4979–4991.

8. ENCODE Project Consortium: **An integrated encyclopedia of DNA elements in the human genome**. *Nature* 2012, **489**:57–74.

9. Bi C, Rogan PK: **Determining Thresholds for Binding Site Sequence Models Using Information Theory**. 2005.

10. Blankenberg D, Von Kuster G, Coraor N, Ananda G, Lazarus R, Mangan M, Nekrutenko A, Taylor J: **Galaxy: a web-based genome analysis tool for experimentalists**. *Curr Protoc Mol Biol Ed Frederick M Ausubel Al* 2010, **Chapter 19**:Unit 19.10.1–21.

11. Giardine B, Riemer C, Hardison RC, Burhans R, Elnitski L, Shah P, Zhang Y, Blankenberg D, Albert I, Taylor J, Miller W, Kent WJ, Nekrutenko A: **Galaxy: A platform for interactive large-scale genome analysis**. *Genome Res* 2005, **15**:1451–1455.

12. Goecks J, Nekrutenko A, Taylor J, $author.lastName $author firstName: **Galaxy: a comprehensive approach for supporting accessible, reproducible, and transparent computational research in the life sciences**. *Genome Biol* 2010, **11**:R86.

13. Cook KB, Kazan H, Zuberi K, Morris Q, Hughes TR: **RBPDB: a database of RNA-binding specificities**. *Nucleic Acids Res* 2011, **39**(Database issue):D301–8.

14. Weirauch MT, Yang A, Albu M, Cote AG, Montenegro-Montero A, Drewe P, Najafabadi HS, Lambert SA, Mann I, Cook K, Zheng H, Goity A, van Bakel H, Lozano J-C, Galli M, Lewsey MG, Huang E, Mukherjee T, Chen X, Reece-Hoyes JS, Govindarajan S, Shaulsky G, Walhout AJM, Bouget F-Y, Ratsch G, Larrondo LF, Ecker JR, Hughes TR: **Determination and inference of eukaryotic transcription factor sequence specificity**. *Cell* 2014, **158**:1431–1443.

15. Ray D, Kazan H, Cook KB, Weirauch MT, Najafabadi HS, Li X, Gueroussov S, Albu M, Zheng H, Yang A, Na H, Irimia M, Matzat LH, Dale RK, Smith SA, Yarosh CA, Kelly SM, Nabet B, Mecenas D, Li W, Laishram RS, Qiao M, Lipshitz HD, Piano F, Corbett AH, Carstens RP, Frey BJ, Anderson RA, Lynch KW, Penalva LOF, et al.: **A compendium of RNA-binding motifs for decoding gene regulation**. *Nature* 2013, **499**:172–177.

16. Ardlie KG, Deluca DS, Segrè AV, Sullivan TJ, Young TR, Gelfand ET, Trowbridge CA, Maller JB, Tukiainen T, Lek M, Ward LD, Kheradpour P, Iriarte B, Meng Y, Palmer CD, Esko T, Winckler W, Hirschhorn JN, Kellis M, MacArthur DG, Getz G, Shabalin AA, Li G, Zhou Y-H, Nobel AB, Rusyn I, Wright FA, Lappalainen T, Ferreira PG, Ongen H, et al.: **The Genotype-Tissue Expression (GTEx) pilot analysis: Multitissue gene regulation in humans**. *Science* 2015, **348**:648–660.

**
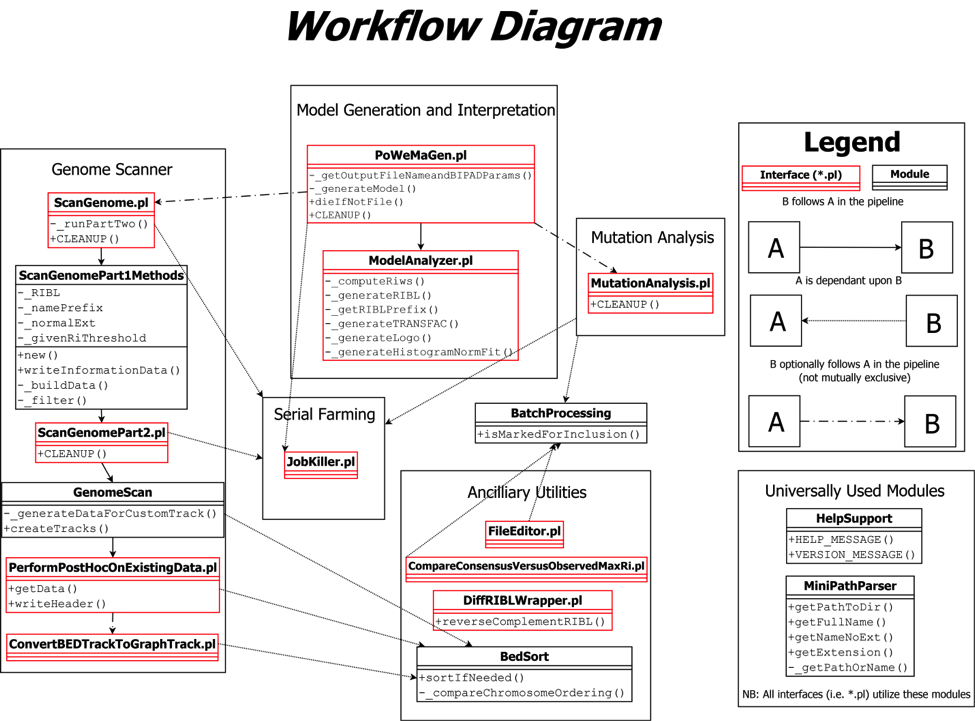
**

**Figure SM1. Diagram of the PWM Generator.** UML Diagram of the distributed processing system. The modules shown call the Bipad and Shannon pipeline C++ engines to perform entropy minimization and genome scanning. Solid arrows indicate embedded capabilities called by the preceding software module; discontinuous arrows indicate separate programs with compatible input/outputs. Ancillary utilities are used for post-processing of output from main programs to compare different models and sort results from GenomeScan output.
