# Supplementary material for: A unified analytic framework for prioritization of non-coding variants of uncertain significance in heritable breast and ovarian cancer
Source: BMC Med Genomics. 2016 Apr 11;9:19. doi: 10.1186/s12920-016-0178-5 (PMC4828881; doi:10.1186/s12920-016-0178-5)
Supplement: Additional file 2: — Provincial Eligibility Criteria. Risk Categories for Individuals Eligible for Screening for a Genetic Susceptibility to Breast or Ovarian Cancer as determined by the Ontario Ministry of Health and Long Tern-Care Referral Criteria for Genetic Counseling (PDF 10 kb) [file 12920_2016_178_MOESM2_ESM.pdf]

Additional File 2 - Risk Categories for Individuals Eligible for Screening for a Genetic Susceptibility to Breast or Ovarian Cancers as determined by the Ontario Ministry of Health and Long Term-Care Referral Criteria for Genetic Counseling

Risk Category	Description
1	Ashkenazi Jewish and BC <50 years, or OC at any age
2	BC <35 years of age
3	Male BC
4	Invasive serous ovarian cancer at any age
5	BC <60 year, and a 1st or 2nd-degree relative with OC or male BC
6	BC and OC in the same individual, or bilateral BC with the first case <50 years
7	Two cases of OC, both <50 years, in 1st or 2nd-degree relatives
8	Two cases of OC, any age, in 1st or 2nd-degree relatives
9	Ashkenazi Jewish and BC at any age, and any family history of BC or OC
10	Three or more cases of BC or OC at any age
11	Relative of an individual with known *BRCA1* or *BRCA2* mutation
12	Ashkenazi Jewish and 1st or 2nd-degree relative or individual with: BC <50 years, or OC at any age, or male BC, or BC, any age, with positive family history of BC or OC.
13	A pedigree strongly suggestive of HBOC, i.e. risk of carrying a mutation for the individual being tested is >10%

Only patients from groups 5-8, and 10 were considered for this study.
