# Supplementary material for: A unified analytic framework for prioritization of non-coding variants of uncertain significance in heritable breast and ovarian cancer
Source: BMC Med Genomics. 2016 Apr 11;9:19. doi: 10.1186/s12920-016-0178-5 (PMC4828881; doi:10.1186/s12920-016-0178-5)
Supplement: Additional file 6: Figure S1. — BRCA1 Deletion Inaccurately Aligned by CASAVA (PDF 22 kb) [file 12920_2016_178_MOESM6_ESM.pdf]

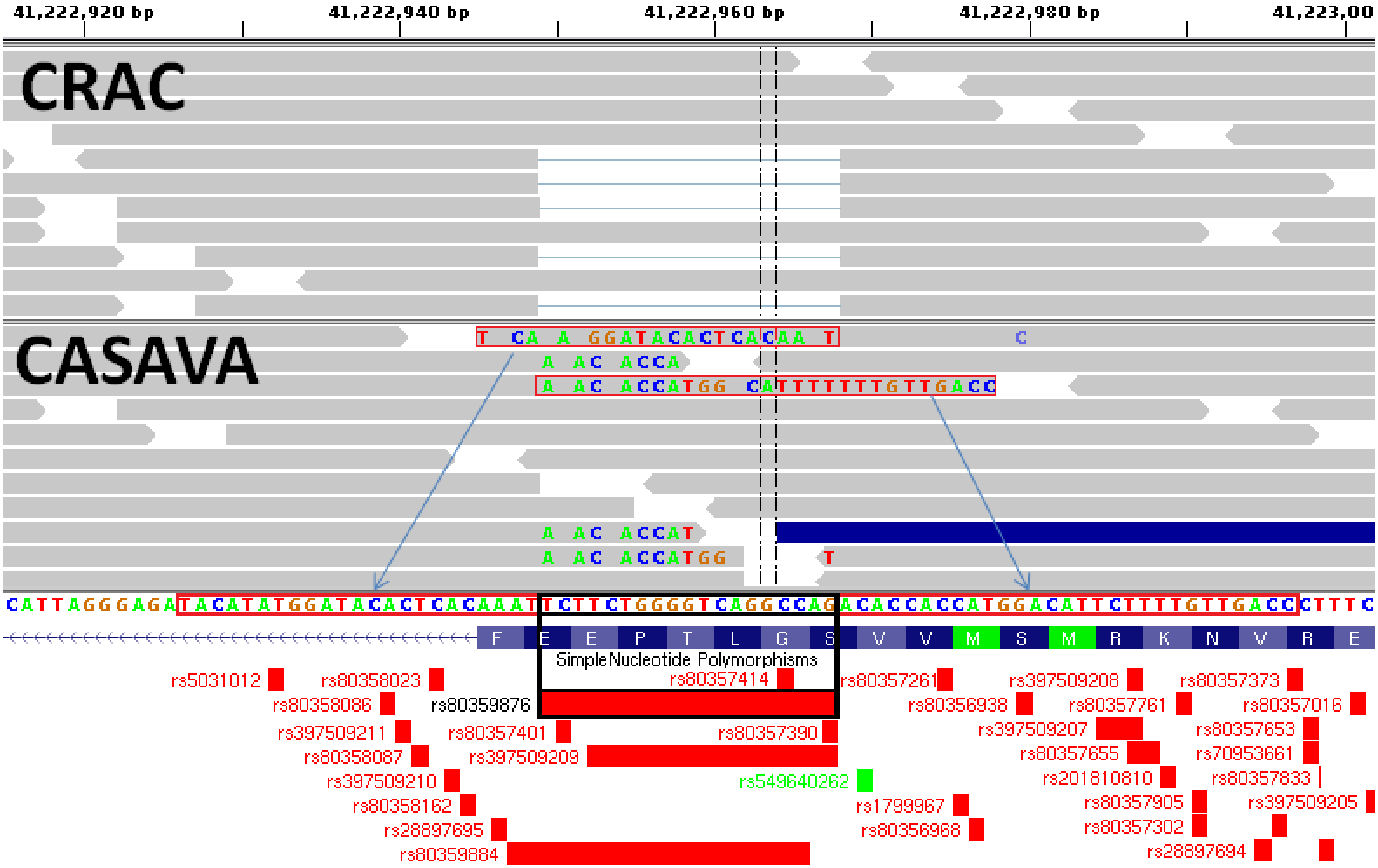

Figure S1. ***BRCA1*** **Deletion Inaccurately Aligned by CASAVA**

A 19 nt *BRCA1* deletion was not identified by one alignment program (CASAVA), and instead appears as a series of SNVs. The *BRCA1* deletion is still observable on IGV (middle box) as these SNVs align to two separate regions of *BRCA1* exon 15 (arrowed red boxes). This deletion leads to a frameshift starting with p.1655Ser and a premature STOP at p.1670, resulting in the loss of 193 AA.
